# OGP46 Induces Differentiation of Acute Myeloid Leukemia Cells via Different Optimal Signaling Pathways

**DOI:** 10.3389/fcell.2021.652972

**Published:** 2021-03-04

**Authors:** Min Zhao, Jiangyun Wang, Mei Qu, Yao Zhao, Haihua Wang, Yu Ke, Ying Liu, Zi-Ning Lei, Hong-Min Liu, Zhenbo Hu, Liuya Wei, Zhe-Sheng Chen

**Affiliations:** ^1^Laboratory for Stem Cell and Regenerative Medicine, Affiliated Hospital of Weifang Medical University, Weifang, China; ^2^School of Pharmacy, Weifang Medical University, Weifang, China; ^3^School of Pharmacy, Zhengzhou University, Zhengzhou, China; ^4^Department of Pharmaceutical Sciences, College of Pharmacy and Health Sciences, St. John’s University, New York, NY, United States; ^5^School of Public Health, Guangzhou Medical University, Guangzhou, China

**Keywords:** acute myeloid leukemia, acute promyelocytic leukemia, differentiation therapy, AML cells with t(8;21) translocation, PML-RARα depletion

## Abstract

Acute myelogenous leukemia (AML) is characterized by blockage of cell differentiation leading to the accumulation of immature cells, which is the most prevalent form of acute leukemia in adults. It is well known that all-trans retinoic acid (ATRA) and arsenic trioxide (ATO) are the preferred drugs for acute promyelocytic leukemia (APL). However, they can lead to irreversible resistance which may be responsible for clinical failure after complete remission (CR). Moreover, the differentiation therapy of ATRA-based treatment has not been effective against AML with t(8;21) translocation. Here we aimed to identify the differentiation effect of OGP46 on AML cell lines (HL-60, NB4, and Kasumi-1) and explore its possible mechanisms. We found that OGP46 has significant inhibitory activity against these cells by triggering cell differentiation with cell-cycle exit at G1/G0 and inhibited the colony-formation capacity of the AML cells. It was shown that OGP46 induced the differentiation of NB4 cells via the transcriptional misregulation in cancer signaling pathway by PML-RARα depletion, while it was attributed to the hematopoietic cell lineage and phagosome pathway in Kasumi-1 cells, which are all critical pathways in cell differentiation. These results highlight that OGP46 is an active agent not only in the APL cell line NB4 but also in AML-M2 cell lines, especially Kasumi-1 with t(8;21) translocation. Therefore, OGP46 may be a potential compound for surmounting the differentiation blockage in AML.

## Introduction

Leukemia is a disease of malignant hematopoietic stem cells with abnormality of cells, which are inhibited differentiation and unrestricted rapidly proliferation. Acute myelogenous leukemia (AML) represents a typical example of a type of leukemia that is characterized by a blockage of differentiation. It is observed in 15–20% of the acute leukemia (AL) in children and approximately 80% of AL in adults (Pui, 1995; De Kouchkovsky and Abdul-Hay, 2016). Acute promyelocytic leukemia (APL), the M3 subtype of AML, is characterized by an increase of abnormal promyelocytic cells in the bone marrow (Warrell et al., 1993; Grignani et al., 1994). APL is one of the most aggressive types and accounts for 10–15% of AML. 95% of APL have t(15;17) (de Thé et al., 1990; Kakizuka et al., 1991) chromosomal translocations generating promyelocytic leukemia/retinoic acid receptor α (PML/RARα) fusion genes (Kitareewan et al., 2002; Shah et al., 2008). Hence, the fusion gene expression is associated with leukemogenesis (Grignani et al., 1993). Moreover, a study has shown that the fusion protein imposes a block to normal differentiation (Grignani et al., 1996). APL is unique model treated with the differentiation inducer, all-trans retinoic acid (ATRA), which can lead to degradation of PML-RARα onco-protein (Yoshida et al., 1996; Nervi et al., 1998). ATRA has transformed APL from being highly fatal to being highly curable (Huang et al., 1988; Wang and Chen, 2008), and the combination of ATRA and arsenic trioxide (ATO) yields a 90% disease-free survival rate at 4 years in APL patients (Shen et al., 1997). However, the development of treatment resistance may occur and different conditions will complicate the healing of APL patients undergoing the ATRA/ATO therapy (Noguera et al., 2019). Therefore, discovering new differentiation inducers against PML/RARα is urgently needed.

The (8;21) translocation, which is associated with 40–80% of M2 subtype of AML and 12–20% of all cases of AML, involves the AML1 (RUNX1) gene on chromosome 21, and the eight-twenty-one (ETO) gene on chromosome 8 (Zhou et al., 2007). This translocation creates the AML1-ETO fusion oncoprotein, which is considered the most common structural chromosomal aberrations in patients with AML (Linggi et al., 2002). Clinically, aggressive cytosine arabinoside (Ara-C) containing chemotherapy is the standard protocol for the treatment of AML with t(8;21). Although Ara-C can induce the complete remission (CR) of more than 60% in non-elderly patients, severe myelosuppression is one of the main causes of death and prolonged hospitalization period in AML patients (Papaemmanuil et al., 2016). Moreover, post-remission chemotherapy consisting of three to four courses of Ara-C followed by chemotherapy is essential in the patients who achieve a first CR, lest they develop recurrent disease (Schlenk, 2014). However, continuous chemotherapy may lead to toxicities and inhibit patients’ immune response. Therefore, the development of innovative therapies is needed to improve patient outcomes and provide alternative therapeutic for t(8;21) AML.

OGP46, a kaurene diterpenoid, was designed and synthesized from Oridonin (Liu et al., 2011). Oridonin was a component of the *Rabdosia rubescens*, a traditional Chinese Medicine, which was effective on 31 cases of primary liver cancer by the clinical observation. As described in our previous work (Wei et al., 2020), OGP46 has been shown to induce cell differentiation and inhibit the cell proliferation of chronic myeloid leukemia (CML) cell lines expressing native BCR-ABL and mutant BCR-ABL, including T315I through the BCR-ABL/JAKSTAT pathway by depleting the BCR-ABL oncogene. Moreover, OGP46 was less toxic to normal blood mononuclear cells. In this report, we evaluated whether OGP46 has significant activity against APL cells and M2 subtype of AML cells, especially t(8;21) translocation by cell differentiation and the possible mechanism behind the activity.

## Results

### OGP46 Significantly Inhibits Proliferation of NB4, Kasumi-1, and HL-60 Cells

**FIGURE 1:**
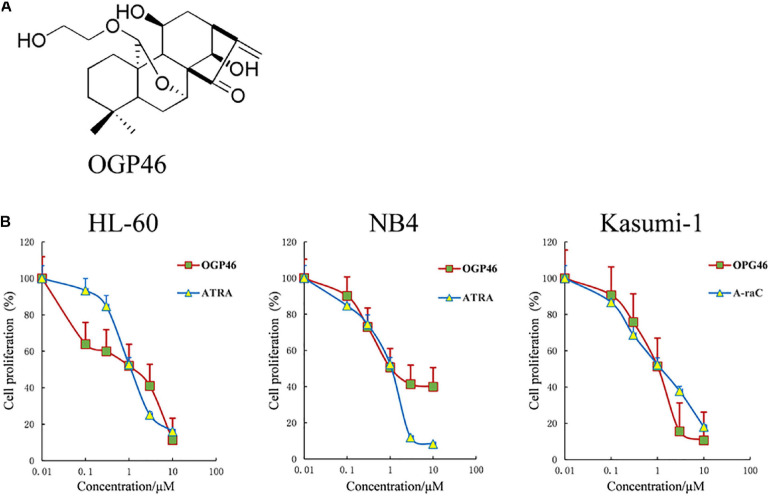
OGP46 has anti- proliferation of HL-60, NB4, and Kasumi-1 cells. **(A)** OGP46’s chemical structure. **(B)** The cell proliferation effect of OGP46 on the AML cells (HL-60, NB4, and Kasumi-1) cell. Cells were treated with OGP46, ATRA, or Ara-C (0.01–10 μM) for 96 h and then determined by CCK-8 assay. The points represent the mean and error bars represent standard error from three independent triplicate experiments.

To estimate the anti-proliferation effect of OGP46 on HL-60, NB4, and Kasumi-1 cell lines, we performed Cell Counting Kit-8 (CCK-8) assay. As shown in Figure 1B, OGP46 significantly inhibited the proliferation of NB4 and HL-60 cells. The IC_50_ values were 1.06 and 1.36 μM at 96 h, respectively, which were comparable with those of ATRA with 1.20 and 1.12 μM, respectively. Similarly, OGP46 also showed an anti-proliferation effect on Kasumi-1 cells [IC_50_ (OGP46) = 1.05 μM, IC_50_ (Ara-C) = 0.93 μM at 96 h]. The data indicate that OGP46 is apparently effective against not only the APL-derived NB4 cell line but also the AML-M2 cell lines including Kasumi-1 and HL-60.

### OGP46 Induces G1/G0 Arrest in NB4, Kasumi-1, and HL-60 Cells

**FIGURE 2:**
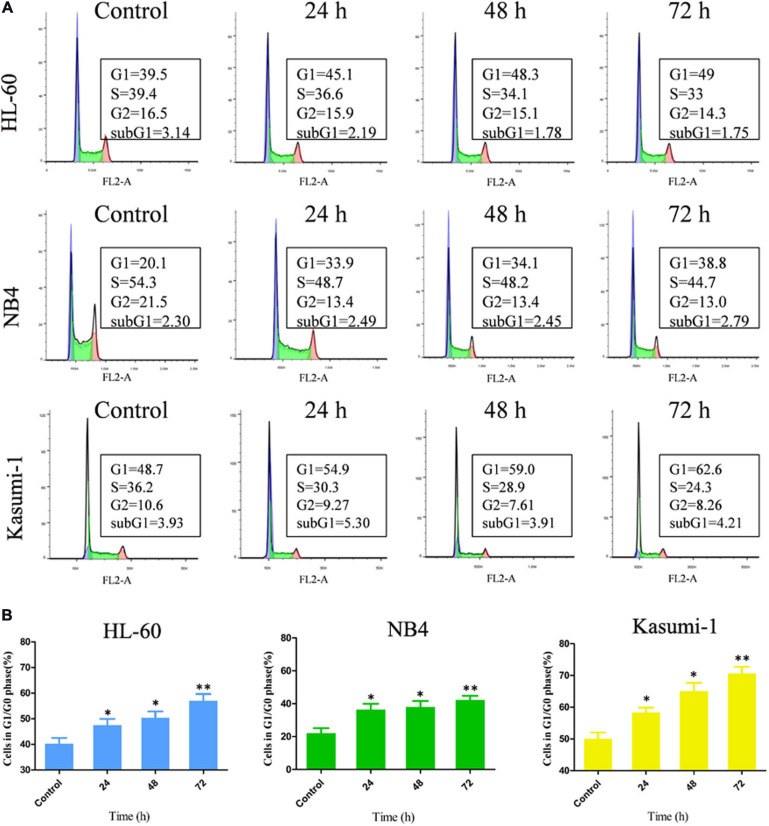
OGP46 induced the cell-cycle exit of NB4, Kasumi-1, and HL-60 cells. **(A)** NB4, HL-60, and Kasumi-1 cells were treated with OGP46 at the concentrations of 1, 2, or 1 μM, respectively, for 24, 48, or 72 h, then stained with PI, and detected by flow cytometry. **(B)** A bar graph showing the percentage of cells at G1/G0 phase a bar graph (*p < 0.05 and **p < 0.01).

To further investigation of the influence of OGP46 on cell cycle distribution in NB4, HL-60, and Kasumi-1 cells, we incubated these cells with 1, 2, or 1 μM OGP46, respectively, for 24, 48, or 72 h. The results showed that the percentage of G1/G0 phase significantly increased within 72 h after OGP46 treatment in these cell lines (Figures 2A,B). These findings reveal that 1 or 2 μM OGP46 could repress cell proliferation by inducing a cell-cycle arrest at G1/G0 in HL-60, NB4, and Kasumi-1 cell lines.

### OGP46 Induces Less Apoptosis in NB4, Kasumi-1, and HL-60 Cells

**FIGURE 3:**
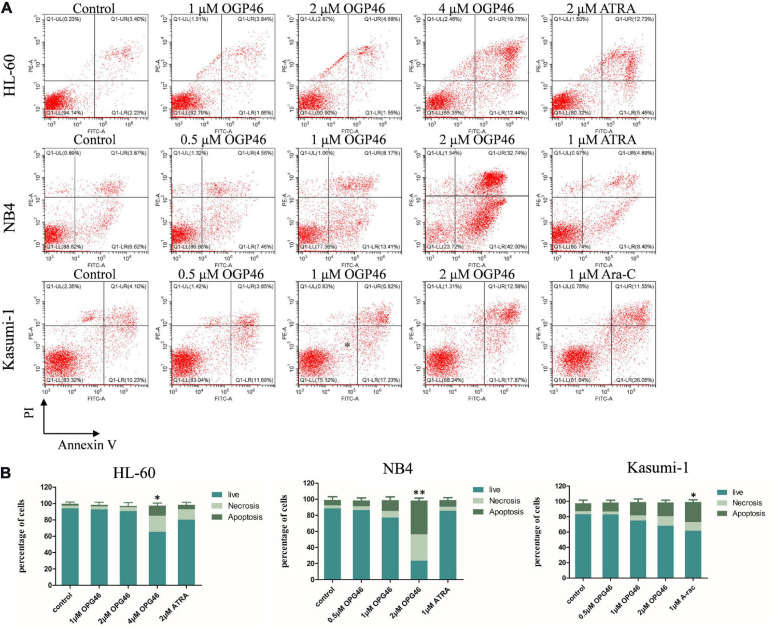
OGP46 induced the apoptosis of HL-60, NB4, and Kasumi-1 cells. **(A)** HL-60, NB4, and Kasumi-1 cell lines were treated with OGP46 (0.5, 1, 2, or 4 μM), ATRA (1 or 2 μM), or Ara-C (1 μM) for 96 h. Cells were then stained with Annexin V-FITC/PI and detected by flow cytometry. **(B)** A bar graph showing the statistical analysis of apoptosis (*p < 0.05 and **p < 0.01).

To determine whether the activity of OGP46 was related to induction of cell apoptosis, HL-60, NB4, and Kasumi-1 cells were incubated with OGP46 (0.5–4 μM) for 96 h. As shown in Figures 1, 3 or 2 μM OGP46 induced less apoptosis in NB4 or HL-60 cells, respectively. Similarly, NB4 or HL-60 cells did not go through obvious apoptosis with ATRA treatment (1 or 2 μM, respectively). In addition, 1 μM OGP46 induced less apoptosis in Kasumi-1 cells. However, Kasumi-1 cells showed enhanced apoptosis induced by the same concentration of Ara-C. The results revealed that the cell-cycle arrest at G1/G0 in these cell lines treated with OGP46 at 1 or 2 μM was not associated with cell apoptosis.

### OGP46 Promotes Differentiation in NB4, Kasumi-1, and HL-60 Cells

**FIGURE 4:**
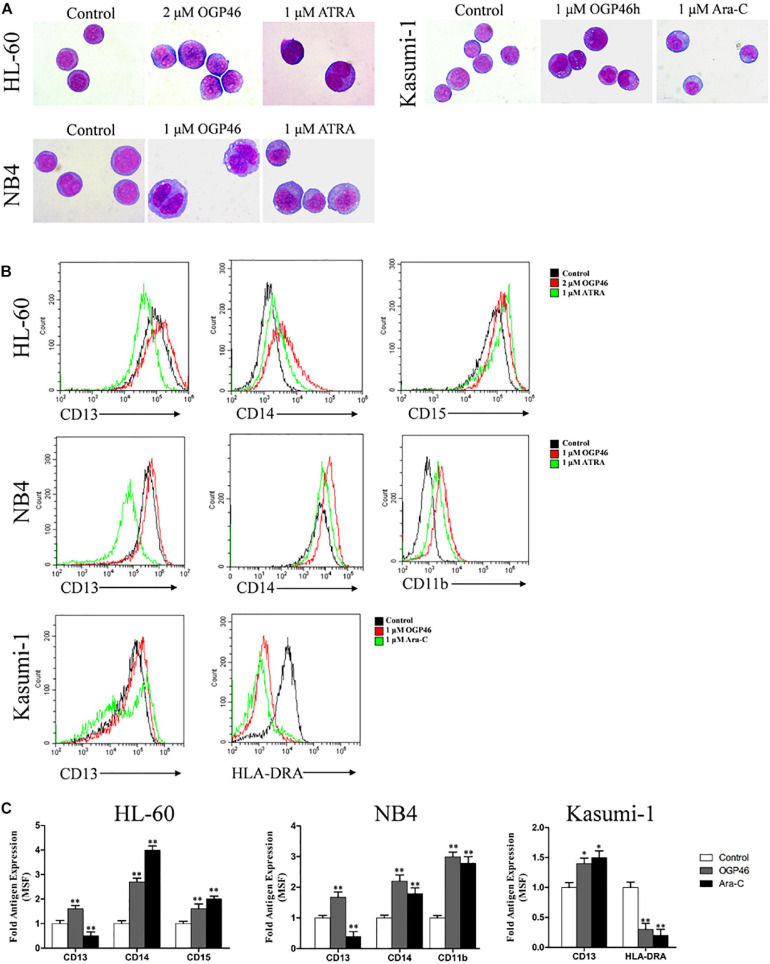
OGP46 promoted cell differentiation of HL-60, NB4, and Kasumi-1 cells. **(A)** Morphological pictures of HL-60, NB4, and Kasumi-1 cell lines were captured by oil immersion lens (×1000). **(B,C)** The expression of cell surface antigens in HL-60, NB4, and Kasumi-1 cells treated with 1, 2, or 1 μM OGP46, respectively, ATRA (1 μM), or Ara-C (1 μM) for 96 h. **(B)** Mean fluorescence intensity (MFI) of antigens. **(C)** A bar graph showing the statistical analysis of MFI (*p < 0.05 and **p < 0.01).

For proliferation inhibition of HL-60, NB4, and Kasumi-1 cells by OGP46 without inducing apoptosis, we performed morphology analysis and cell surface antigen expression analysis to determine the cell differentiation effect of OGP46 on these cells. It can be seen from Figure 4A that HL-60, NB4, and Kasumi-1 cells undergo morphological changes, such as polyploidization, increase of cell size, and decrease of the proportion of nucleus to cytoplasm. The changes of these cells treated with OGP46 were similar to the known effects of ATRA but not of Ara-C. Moreover, it is shown in Figures 4B,C that OGP46 at 1 μM significantly up-regulated the expression of cell surface antigens CD11b, CD13, and CD14 (myeloid differentiation markers) in the NB4 cell line. Similarly, 2 μM OGP46 induced cell differentiation of the HL-60 cell line, as evidenced by up-regulation of the myeloid differentiation markers CD13, CD14, and CD15. In addition, 1 μM OGP46 also increased the expression of CD13, with a significant decrease of HLA-DRA [immune regulation antigen, one of the major histocompatibility complexes Class II (MHCII)] in Kasumi-1 cell line. Thus, our findings indicate that the G1/G0 arrest of cell-cycle perhaps due to the cell differentiation induced by OGP46.

### OGP46 Remarkably Suppressed Colony-Formation Capacity in NB4, Kasumi-1, and HL-60 Cells

**FIGURE 5:**
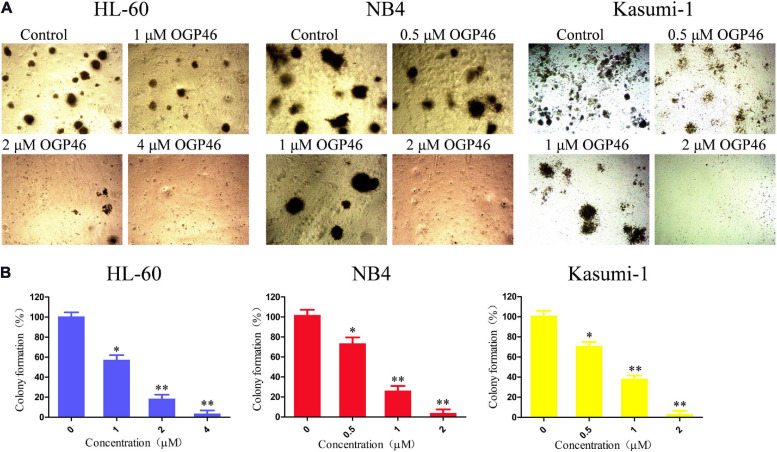
OGP46 reduced the colony-forming efficiency of HL-60, NB4, and Kasumi-1 cells. **(A)** HL-60, NB4, and Kasumi-1 cells were incubated with OGP46 (0.5–4 μM) for 15 days and then examined with light microscopy. **(B)** A bar graph showing the statistical analysis of colony-formation number (*p < 0.05 and **p < 0.01).

We assessed the effect of OGP46 on the colony formation of HL-60, NB4, and Kasumi-1 cells. It can be seen from Figures 5A,B that the number of colonies was decreased significantly with increasing concentration of OGP46. Moreover, the cells were not able to form colonies in these cell lines treated with OGP46 at 2 or 4 μM, as the differentiated cells that have lost their capability to form colonies. The results indicate that OGP46 could significantly inhibit the colony-formation ability of these cells.

### Signaling Pathways Associated With Cell Differentiation Induced by OGP46 in NB4 and Kasumi-1 Cells

**FIGURE 6:**
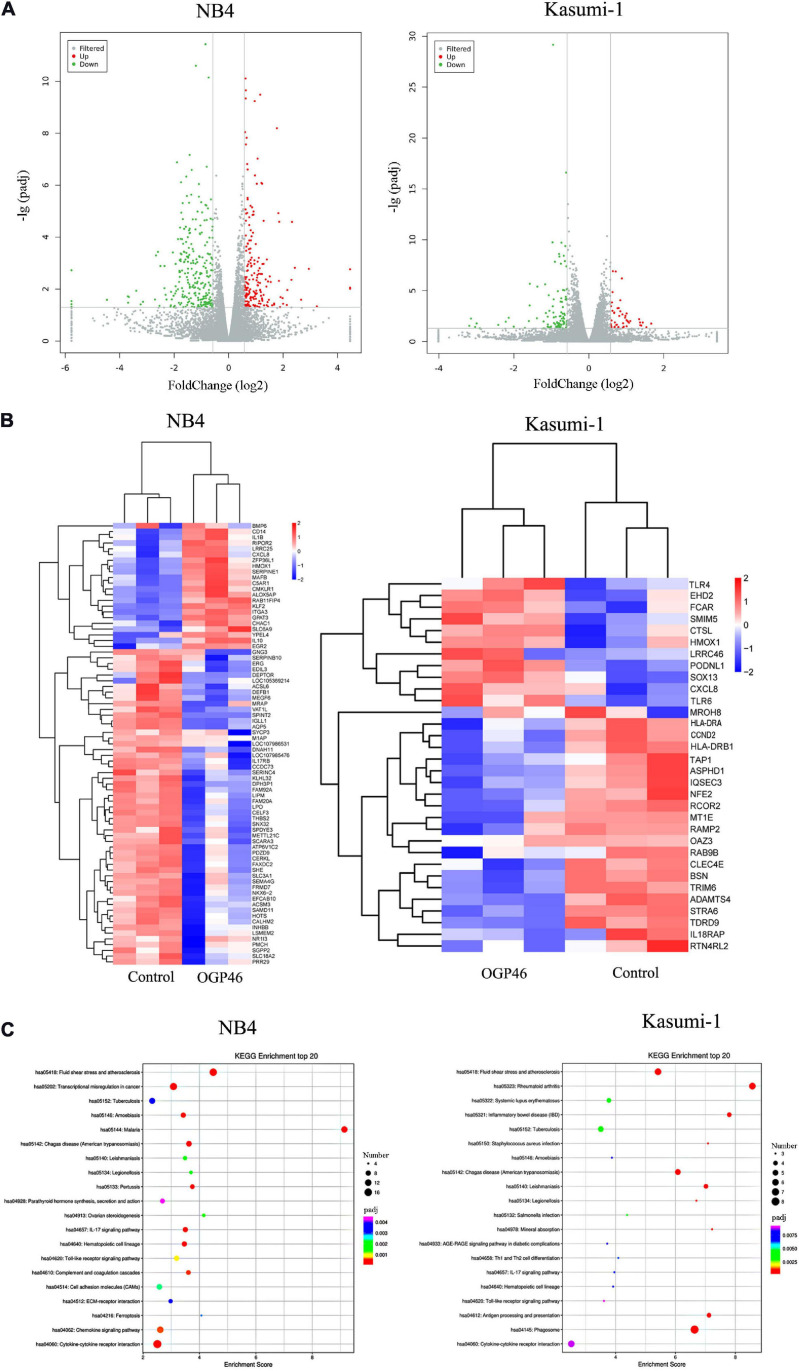
The effect of OGP46 is associated with the transcriptional misregulation in cancer pathway in NB4 cells and hematopoietic cell lineage, and phagosome pathways in Kasumi-1 cells. **(A)** Volcano plots of NB4 and Kasumi-1 cells. **(B)** Heatmap of differentially expressed genes [only 100 genes (*p* < 0.05 and | log _2_ FC| > 2) according to their *p*-value in the NB4 cell line and 41 genes (*p* < 0.05 and | log _2_ FC| > 2) according to their *p*-value in the Kasumi-1 cell line are shown in this figure]. **(C)** KEGG pathway analyses of all differentially expressed genes. NB4 or Kasumi-1 cells were incubated with 1 μM OGP46 for 72 h, and then mRNA sequencing was performed. The figures are representative of three independent experiments.

To discover major mechanism regulating the induction differentiation by OGP46, the mRNA expression levels using mRNA sequencing (mRNA-seq) were investigated in NB4 and Kasumi-1 cell lines incubated with OGP46. As shown in Figure 6A, we screened for 182 genes that were up-regulated, while 258 genes were down-regulated in NB4 cells. Similarly, there were 47 genes with significant up-regulation, whereas 96 genes were down-regulated in Kasumi-1 cells. These data suggest that OGP46 does not affect all genes universally for its different effects on the mRNA expression of various species. As shown in Figure 6B, genes such as CD14, ITGA3 (CD49C), CXCL8, IL1B, and IL10 were significantly up-regulated, whereas genes including IL17RB and INHBB were significantly down-regulated in NB4 cells incubated with OGP46. Similarly, OGP46 significantly up-regulated the expression of genes including CXCL8, TLR4, TLR6, HMOX1, CTSL, and FCAR in Kasuimi-1 cells. In addition, the expression of genes such as HLA-DRA, HLA-DRB1, TAP1, CCND2 (cyclinD2), and IL18RAP was significantly reduced after OGP46 treatment. Furthermore, it can be seen from Figure 6C, by the Kyoto Encyclopedia of Genes and Genomes (KEGG) analysis, several key signaling pathways, such as transcriptional misregulation in cancer pathway [CXCL8, CD14, cyclin-dependent kinase inhibitor 1A (CDKN1A), ITGAM (CD11b), ID2, BCL6, ITGB7, MMP9, CEBPB, ERG, and TFE3 were enriched), cytokine–cytokine receptor interaction pathway (CXCL8, IL10, IL1R1, IL1B, INHBB, CCR2, IL17RB, CXCL16, CCL2, TNFSF9, BMP2, and CSF2RA were enriched), which were associated with the differentiation of NB4 cells. In addition, the hematopoietic cell lineage (HLA-DRA, HLA-DRB1, CD13, and GP1BB), phagosome pathway (HLA-DRA, HLA-DRB1, TAP1, CTSL, and FCAR were enriched), and cytokine–cytokine receptor interaction pathway (CXCL8, IL18RAP, IL17RE, CXCL1, and ACVR2A were enriched) which were also related to the cell differentiation of Kasuimi-1 cells. Our finding also revealed that cytokine–cytokine receptor interaction pathway was common in both NB4 and Kasumi-1 cell lines.

**FIGURE 7:**
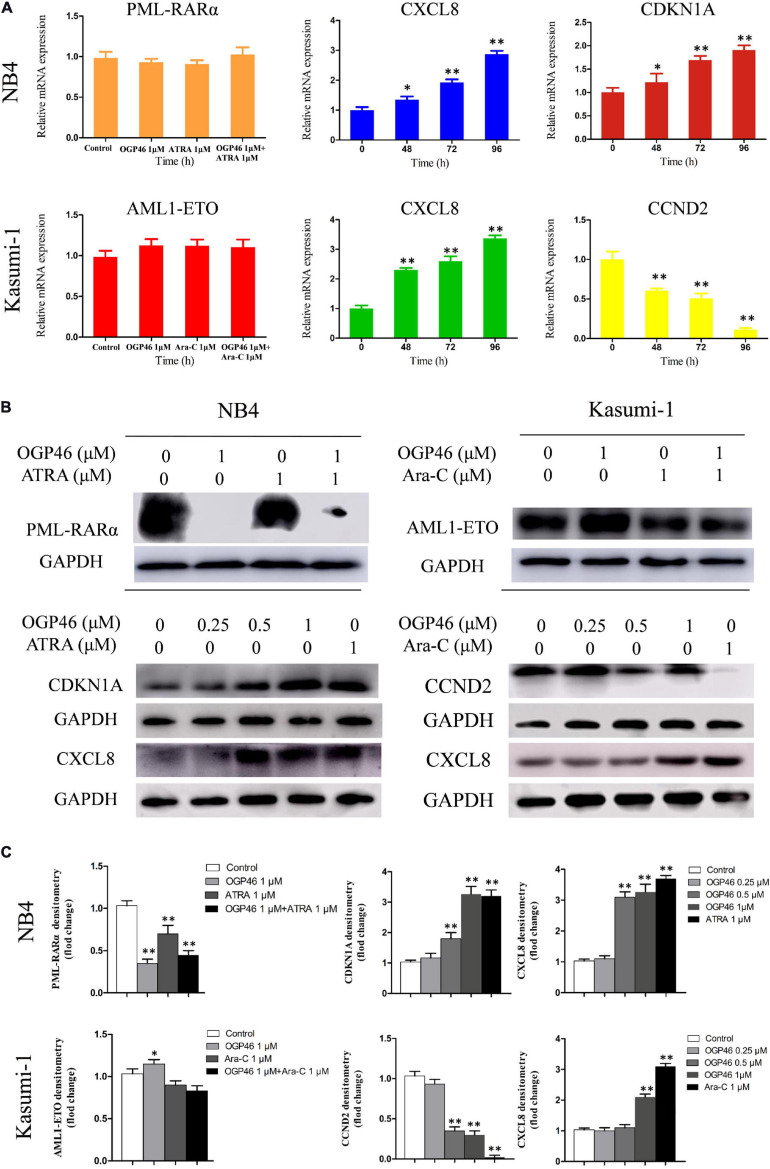
The expression of mRNA and protein of PML-RARα, AML1-ETO, CXCL8, CDKN1A, and CCND2 related to the cell differentiation or cell cycle arrest. **(A,B)** Real-time PCR and Western blotting were used to determine the mRNA and protein expression in NB4 and Kasumi-1 cell lines incubated with OGP46. NB4 or Kasumi-1 cells were treated with OGP46 (1 μM) with or without ATRA (1 μM) or Ara-C (1 μM) for 96 h for detection of PML-RARα and AML1-ETO. NB4 or Kasumi-1 cells were treated with OGP46 (1 μM), ATRA (1 μM), or Ara-C (1 μM) for the indicated time for detection of CXCL8, CDKN1A, and CCND2. **(C)** Protein expression was quantified by the software AI600 images. *p < 0.05 and **p < 0.01.

RNA-seq results were verified, by real-time PCR and Western blotting in NB4 and Kasumi-1 cells. It can be seen from Figures 7A–C that the transcriptional and protein levels of CDKN1A and CXCL8 were up-regulated significantly, while the expression of CCND2 at both levels was remarkably down-regulated, which was consistent with the result of mRNA-seq.

Because PML-RARα is a critical fusion gene in the NB4 cell line and it is involved in the transcriptional misregulation in cancer pathway (KEGG pathway hsa05202), we next investigated the effect of OGP46 on transcriptional and protein level of PML-RARα in NB4 cells. The results showed that PML-RARα was significantly inhibited at the protein level rather than the transcriptional level (Figures 7A–C). These data reveal that the inhibition efficiency of OGP46 on NB4 cells perhaps was contributed to the depletion of the PML-RARα protein. Similarly, considering that AML1-ETO involved in the transcriptional misregulation in cancer pathway plays central roles in Kasuimi-1 cells, the transcriptional and protein levels of AML1-ETO were determined. It can be seen from Figures 7A–C that OGP46 did not alter the expression of mRNA and protein levels of AML1-ETO. These results also indicate that the effects of OGP46 on the PML-RARα and the AML1-ETO protein are different.

## Discussion

Acute myelogenous leukemia is a complex malignant disease that is characterized by myeloid cell differentiation blockage (Olsson et al., 1996; Vradii et al., 2005), which was classified into M0–M7 subtypes. APL, AML subtype M3, is characterized by the presence of the leukemogenic PML-RARα fusion gene, representing 5–10% of AML cases. In addition, the (8;21) is one of the most frequent chromosomal translocations observed in 12–20% of all AML patients (Spirin et al., 2014) and 40–80% of AML-M2-subtype. For the past few decades, the standard therapy of AML has been based on the combination of an anthracycline and Ara-C (Bjerre et al., 2020). Although CR rates are initially high, the adult 5-year overall survival rate of AML patients is less than 40% due to chemotherapy resistance (Olsson et al., 1996; Wang and Chen, 2008). Fortunately, the differentiation therapy of ATRA dramatically improves long-term clinical outcomes for APL patients (Tallman et al., 2002; Hu et al., 2009). However, the development of treatment resistance may also occur in APL patients. Moreover, clinical effectiveness of ATRA-based differentiation therapy has no effects on the non-APL AML (Gocek and Marcinkowska, 2011). Therefore, in this study, we investigated the differentiation of AML cell lines including APL cells and M2 subtype AML cells, using a diterpenoid compound, OGP46.

Our previous study had found that OGP46 exhibited potent activity against CML cell lines by inducing cell differentiation (Wei et al., 2020). In this study, we discovered that OGP46 induced cell-cycle arrest at the G1/G0 phase and caused inhibition of cell proliferation by inducing cell differentiation in HL-60, NB4, and Kasumi-1 cells. Furthermore, the effect of OGP46 may be attributed to the transcriptional misregulation in cancer pathway in the NB4 cell line. In addition, hematopoietic cell lineage and phagosome pathways play central roles in the differentiation of Kasumi-1 cell line. Altogether, we found that OGP46 is effective against AML cell lines by inducing cell differentiation through different signaling pathways depending on cell types.

The transcriptional misregulation in cancer pathway is involved in PML-RARα, which has been found responsible for the differentiation block (Zhu et al., 1999). Thus, PML-RARα plays an important role in cell differentiation in the transcriptional misregulation in cancer pathway. Here, we showed that OGP46 had the cell differentiation effect on NB4 cells associated with the transcriptional misregulation in cancer pathway. Moreover, PML-RARα protein expression was inhibited by OGP46. These results reveal that the differentiation of NB4 cells induced by OGP46 originated from transcriptional misregulation in cancer pathway by inhibiting the expression of PML-RARα protein. In contrast, OGP46 did not alter the expression of mRNA and protein levels of AML1-ETO in Kasumi-1 cell line, which involved in the transcriptional misregulation in cancer pathway. It can be concluded that the induction of cell differentiation by OGP46 was not due to the transcriptional misregulation in cancer pathway in Kasumi-1 cell line.

Hematopoietic cell lineage, a differentiation-related pathway, refers to the developmental of the hematopoietic cells’ differentiation into various hematopoietic lineages such as macrophages and erythrocytes granulocytes (Zhang et al., 2017). The current study found that OGP46 treatment promoted cell differentiation with morphological changes and decreasing HLA-DRA, with an increase in the expression of the cell surface antigen CD13 (Figure 4) in Kasumi-1 cell line. Therefore, these results indicate that OGP46-mediated differentiation of Kasumi-1 cells may be related to hematopoietic cell lineage pathway via changes in expression of cell surface antigens.

It is reported that phagosome pathway is the most optimal and common in both Kasumi-1 and MV4-11 AML cell lines induced by differentiation inducers, Trichostatin A and 5-Azacytidine (Asmaa et al., 2020). Moreover, the phagosome pathway is involved in the MHC II and antigen processing and presentation (KEGG map^1^). In this study, we showed that OGP46 had inhibitory activity against Kasumi-1 cells. Combined with the KEGG enrichment pathways of differential expression genes, it was concluded that immune-related signaling pathways such as phagosome were enriched. Moreover, the increasing expression of CD13 (macrophage/monocyte marker) (Figures 4B,C) suggested the involvement of macrophages/monocyte as the main phagocyte during enteritis in the phagosome pathway. Therefore, it may be concluded that OGP46 induces macrophage differentiation of Kasumi-1 cells, which activates the phagosome pathway.

Interleukins (ILs), a group of cytokines, are secreted by various immune cell types such as macrophages and T lymphocytes. Here, we found that OGP46 induced cell differentiation with up-regulation of CXCL8, as well as ATRA induced CXCL8 production in NB4 and Kasumi-1 cells (Figures 7A–C). This result is in accordance with the report that differentiation induction by ATRA and/or ATO leads to a marked up-regulation of chemokine expression in APL cells (Luesink et al., 2009). The data reveal that OGP46 may induce chemokine production in differentiating leukemic cells. Hence, the common cytokine–cytokine receptor interaction pathway is activated by the induction of cell differentiation but not the signaling pathways which regulates the cell differentiation induced by OGP46 in both NB4 and Kasumi-1 cell lines.

Thus, it was shown that the induction of cell differentiation by OGP46 may be attributed to the transcriptional misregulation in cancer pathway in the NB4 cell line, while it is originated from hematopoietic cell lineage and phagosome pathways in the Kasumi-1 cell line. These were due to their different molecular characteristics (PML-RARα vs AML1-ETO), as evidenced by the result that OGP46 treatment caused the inhibition of PML-RARα expression at protein level, while it did not affect the AML1-ETO expression at both the mRNA and protein levels (Figures 7A–C).

It is well known that cell differentiation and cell-cycle arrest appear to be coupled (Hass et al., 1992). CDKN1A plays an important role in cell cycle. Moreover, it is involved in cell proliferation and differentiation (Kreis et al., 2019). Similarly, CCND2 is one of the cyclins which plays a key role in cell-cycle regulation and differentiation (Li et al., 2016). Our results showed that OGP46 caused cell-cycle arrest at G1/G0, and also induced cell differentiation in AML cells (Figure 2). Moreover, OGP46 treatment up-regulated the expression of CDKN1A at mRNA and protein levels and down-regulated the expression of CCND2 at both levels (Figures 7A–C). Thus, the cell-cycle exit at G1/G0 phase may be due to the activation of CDKN1A or inhibition of CCND2, observed by mRNA-seq and verified by PCR and Western blotting. These results are in accordance with the findings that up-regulation of CDKN1A and decrease the expression of CCND2 at both mRNA and protein levels caused cell cycle arrest at the G1/G0 phase (Li et al., 2016; Pitrone et al., 2019).

## Conclusion

Our findings show that OGP46 has anti-proliferation effect against not only the APL cell line, NB4, but also the M2 subtype of AML cell lines, including HL-60 and Kasumi-1 with t(8;21) translocation. Moreover, our findings demonstrate that OGP46 induced cell differentiation and inhibited the colony-formation ability of these three cell lines through transcriptional misregulation in cancer pathway via depletion of PML-RARα in NB4 cells. OGP46 also induced cell differentiation and inhibition of the colony-formation ability of cells via hematopoietic cell lineage and phagosome pathways in Kasumi-1 cells. Its ability to overcome the differentiation block in NB4, Kasumi-1, and HL-60 cells makes it a potential compound that merits further study to treat AML patients.

## Materials and Methods

### Chemicals

OGP46 was prepared by Hong-Min Liu’s lab, the structure of which is shown in Figure 1A. OGP46 was dissolved in dimethyl sulfoxide and the stock solutions of OGP46 (10 mM) were stored at −20°C. The OGP46 solutions used in various *in vitro* assays were diluted in RPMI 1640 medium and the corresponding concentrations of DMSO were used as control. Penicillin, fetal bovine serum (FBS), streptomycin, and RPMI-1640 were purchased from Sigma–Aldrich (St. Louis, MO, United States). Propidium iodide (PI)/RNase and fluorescein isothiocyanate (FITC)—Annexin V were purchased from Becton Dickinson (San Diego, CA, United States). Cell Counting Kit-8 was purchased from Solarbio (Beijing). FITC anti-human CD11b (cat #301403), PE anti-CD13 (cat #301704 RRID: AB_314180), FITC anti-CD14 (cat #301804, RRID: AB_314186), and PE anti-CD15 (cat #301906, RRID: AB_314198) were purchased from Biolegend Inc. (San Diego, CA, United States). MethoCult H4435 (cat #04435) and H4100 (cat #04100) were obtained from STEMCELL Technologies (Vancouver, BC, Canada). Monoclonal antibodies against GAPDH (Cat #5174), CDKN1A (Cat #2947), CCND2 (Cat#3741), and AML1-ETO (Cat #4336) were obtained from Cell Signaling Technology (Beverly, MA, United States). Monoclonal antibodies including anti-CXCL8 (ab110727) and anti-PML-RARa (ab43152) were obtained from Abcam (Cambridge, MA, United States). SYBR^®^ Premix Ex Taq^TM^ reagent kit and PrimerScript RT reagent kit were used for real-time PCR (TAKARA Bio, Otsu, Japan).

### Cell Lines and Cell Culture

HL-60 (DSMZ no.: ACC 3, at the time of the discovery of induction differentiation by ATRA, it was believed to be APL, only manifested later that it originate from M2 subtype of AML), NB4 (DSMZ no.: ACC 207, M3 subtype of AML expressing PML-RARα, APL cell line), and Kasumi-1 [DSMZ no.: ACC 220, M2 subtype of AML with t(8;21) translocation expressing AML1-ETO] cell lines were used. All cell lines were maintained in RPMI 1640 medium containing 10% FBS or 20% FBS and 1% streptomycin/penicillin. The culture condition was temperature 37°C and 5% CO_2_.

### Cell Proliferation Assay

Cell proliferation was determined with CCK-8 assay. HL-60, NB4, and Kasumi-1 cells were seeded at about 5000 cells/well, in a 96-well plate and cultured. 24 h later, cells were incubated with OGP46, ATRA, or Ara-C at the various concentrations (0.01–10 μM). The final DMSO concentrations during all incubations were not more than 0.1%, which had no observable toxic effects to cells. Following treatment for 96 h, 10 μL of CCK-8 reagent was supplied to each well and incubated for 4 h. Finally, the absorbance was determined at 450 nm by a microplate reader. Results are expressed as percent of cell viability normalized to DMSO-treated control cells.

### Cell Cycle Analysis

NB4, HL-60, and Kasumi-1 cells were treated with OGP46 (1 or 2 μM) for 24, 48, or 72 h. The cells were collected and fixed with 70% ethanol at −20°C and then stained with PI (50 mg/mL) and RNase A (100 mg/mL) for 30 min in the dark at room temperature. The percentage of cells in G0/G1, S, and G2/M was detected by flow cytometry analysis with a BD FACSCalibur System.

### Cell Apoptotic Rate Analysis

To assess apoptotic rate of cells, HL-60, NB4, and Kasumi-1 cell lines were incubated with OGP46 (0.5–4 μM) for 96 h. After treatment, cells were centrifuged, collected, then washed with cold PBS, resuspended in 1× binding buffer, and stained with Annexin V-FITC/PI. After incubation for 30 min in the dark at room temperature, cell apoptotic rate was quantitatively detected by flow cytometry.

### Analysis of Cell Morphology

HL-60, NB4, and Kasumi-1 cells were incubated with 2, 1, or 1 μM OGP46, respectively, for 96 h. Cells were centrifuged and collected, then slides were made, air dried and fixed with methanol, incubated with Wright-Giemsa for 10 min, and the morphology of the cells was observed under light microscopy.

### Analysis of Cell Surface Antigens

NB4, Kasumi-1, and HL-60 cells were treated with OGP46 (1, 1, or 2 μM, respectively). After 96 h, the cells were centrifuged, collected, then washed, and stained with monoclonal antibodies for 30 min in the dark at room temperature. Finally, after the incubation, cells were collected, washed, and then resuspended in PBS. The cells conjugated with the antibodies were determined with flow cytometry.

### Colony-Formation Assay

About 5000 cells (NB4, Kasumi-1, and HL-60) were incubated with indicated concentrations of OGP46 in 500 mL of 2.6% methyl-cellulose medium supplemented with 10% FBS in each well of 24-well plates. Following 15 days of incubation, the total number of separate colonies consisting of more than 50 cells was counted using an inverted microscope.

### mRNA-Sequencing

mRNA sequencing was carried out on NB4 and Kasumi-1 cell lines. After OGP46 incubation for 48 h, cells were collected for RNA extraction. As described in our previous work (Wei et al., 2020), oligo (dT) selection was used to enrich poly (A) mRNA from total RNA. The cDNA libraries were prepared and then sequenced on an Illumina genome analyzer. The gene expressions were estimated using fragments per kilobase of exon per million fragments mapped (FPKM) values. The false discovery rate (FDR) was used to identify the threshold of the p value to analyze the significance of the differences. Significantly differentially expressed genes (DEGs) were identified with a level [a corrected *p*-value of 0.05 and absolute value of log 2 FC (fold change) ≥ 0.58]. Enrichment analysis of Gene Ontology and KEGG pathway of DEGs was performed with R language. Significantly enriched GO or KEGG pathway was identified with an adjusted *p*-value of <0.05.

### Real-Time PCR

The cDNA was synthesized with a PrimerScript RT reagent kit. Quantitative real-time PCR was performed using SYBR Green PCR mix on an Applied Biosystems 7500 Fast PCR System. The relative expression of mRNA was analyzed by the 2^–△ △^^*Ct*^ method. Primers were used as follows: forward GAPDH: 5′-TGGGTGTGAACCATGAGAAGT-3′ and reverse GAPDH: 5′-TGAGTCCTTCCACGATACCAA-3′; forward CDKN1A: 5′- GTG GGG TTA TCT CTG TGT TAG GG-3′ and reverse CDKN1A: 5′-CCC TGT CCA TAG CCT CTA CTG C- 3′; forward CCND2: 5′-GGACATCCAACCCTACATGC-3′ and reverse CCND2: 5′-CGCACTTCTGTTCCTCACAG-3′; CXCL8: 5′-TGGCAGCCTTCCTGATTTCT-3′ and reverse CXCL8: 5′- GGGTGGAAAGGTTTGGAGTATG-3′; forward PML-RARα: 5′-AAGTGAGGTCTTCCTGCCCAA-3′, reverse PML-RARα: 5′-GGCTGGGCACTATCTCTTCAGA-3′; forward AML1-ETO: 5′-CACCTACCACAGAGCCATCAAA-3′, reverse AML1-ETO: 5′-ATCCACAGGTGAAGTCTGGCATT-3′.

### Western Blotting Analysis

Western blotting was carried out as described (Wei et al., 2020) by using the indicated antibodies.

### Statistical Analysis

All experiments were repeated at least three times. ANOVA was used to determine the statistical significance. *p* < 0.05 or *p* < 0.01 was set as statistical significance.

## Data Availability Statement

The data presented in this study are deposited in the GEO of NCBI, https://www.ncbi.nlm.nih.gov/geo/query/acc.cgi?acc=GSE167084 repository, accession number is GSE167084.

## Author Contributions

MZ: conceptualization, methodology, and writing—original draft. JW: methodology. MQ, YZ, HW, YK, YL, Z-NL, and ZH: methodology. H-ML: supervision. LW: writing—review and editing and supervision. Z-SC: conceptualization and supervision. All authors contributed to the article and approved the submitted version.

## Conflict of Interest

The authors declare that the research was conducted in the absence of any commercial or financial relationships that could be construed as a potential conflict of interest.

## Footnotes

1 http://www.affbiotech.cn/pathway.php?kegg=04145
